# Antimicrobial efficacy of herbal and silver nanoparticles based root canal irrigants

**DOI:** 10.6026/973206300222415

**Published:** 2026-04-30

**Authors:** Kamil Shahnawaz, Anamika Anamika, Swati Sagar, Shakir Alam, Abhijeet Alok

**Affiliations:** 1 Department of Dentistry, Darbhanga Medical College & Hospital, Bihar, India; 2 Department of Oral and Maxillofacial Pathology, Mithla Minority Dental College & Hospital, Darbhanga, Bihar, India

**Keywords:** Antimicrobial efficacy, *E faecalis*, herbal irrigants, silver nanoparticles

## Abstract

The complete removal of organic material and microorganisms from the root canal is essential to the effectiveness of root canal therapy. Root canal irrigants aid in the eradication of root canal microorganisms in addition to mechanical cleaning. Therefore, it is of interest to report/ describe the antibacterial effectiveness of root canal irrigants based on herbal and silver nanoparticles. The test irrigant's impact on *Enterococcus faecalis* and *Staphylococcus aureus* was evaluated in conjunction with a water control group. Silver nanoparticle-based root canal irrigants show the high inhibitory zone against *E. faecalis* and *Staphylococcus aureus* compared to herbal irrigants.

## Background:

The complete removal of microbes and organic debris is essential to the effectiveness of root canal therapy [1]. The predominant pathogens in root canal infections are bacteria. It is widely known that the main cause of pulpal and periapical diseases are the existence of microorganisms in the root canal system. Eliminating these infectious germs is the main goal of all endodontic procedures. Even though the mechanical method makes an effort to debride contaminated canal walls, it is unable to completely eradicate pollution from untreated root canal system regions. Owing of these restrictions, numerous irrigation techniques have been created to enhance and supplement the mechanical debridement process [2]. Chlorhexidine (CHX), sodium hypochlorite (NaOCl), and ethylenediaminetetraacetic acid (EDTA) are the most widely used root canal irrigants [3, 4]. Because of its strong antibacterial qualities and capacity to disintegrate tissue remains, sodium hypochlorite is the recommended option. Although it doesn't dissolve tissue, chlorhexidine has comparable antibacterial activity [5]. Nevertheless, NaOCl tastes bad and can result in edema, ecchymosis, tissue necrosis and paresthesia if applied incorrectly. Other drawbacks include toxicity, a possible caustic impact, and the incapacity to remove the smear layer [6]. As a result, different irrigants were tested. As endodontic irrigants, a variety of herbal extracts are showing promise, such as neem, tulsi extracts, *Aloe vera*, *Morindacitrifolia*, curcuma longa, turmeric, *triphala*, proplis, *Salvadora persica* (Miswak) and *Terminalia chebula* [7]. *triphala* is an Indian Ayurvedic herbal medicine made from the dried and powdered fruits of three medicinal plants: *Terminalia chebula*, *Terminalia bellerica* and *Emblica officinalis*. Its high concentration of citric acid may act as a chelating agent and aid in the removal of the smear layer. It has excellent anti-inflammatory and antibacterial qualities [8]. Turmeric is a therapeutic herb. Curcumin is the primary component in turmeric. Due to its antimicrobial, anti-inflammatory, anti-carcinogenic, nontoxic and analgesic effects, curcumin has been used extensively in dentistry and medicine for centuries [9]. By providing a greater surface area and higher charge density, nanoparticles improve interactions with bacterial surfaces and increase antibacterial activity in root canal therapy [10]. Therefore, it is of interest to assess the antimicrobial efficacy of herbal (turmeric and Triphals) and silver nanoparticles based root canal irrigants.

## Materials and Methods:

This *in vitro* study was done in the department of Conservative dentistry and endodontics. *triphala* powder (IMPCOPS Ltd., Chennai, India) was mixed in 10% dimethylsulfoxide (SD Fine Chemicals, Chennai, India). This is to create an irrigation solution with a 5 mg/ml concentration. Two hundred fifty grams of powdered Curcuma longa rhizomes were combined with one thousand millilitres of pure ethanol in a flask to create a 12.5% turmeric irrigant. The flask was wrapped in aluminum foil and left overnight. The extract was obtained from the ethanol by evaporation after being filtered via filter paper. A 12.5% turmeric solution was made by diluting the roughly 112.5 grams that was produced with distilled water.

## Silver nanoparticle solution preparation:

To create a 0.1% silver nanoparticle solution, 0.1 gm of silver nanoparticles were diluted with 100 mL of distilled water in a falcon tube and put in a sonicator. Micromaster Laboratories Pvt. Ltd. in Thane, India provided the two reference bacterial strains, *Enterococcus faecalis* (ATCC: 29212) and *Staphylococcus aureus* (ATCC: 25923).The two facultative strains were cultured at 37°C for 24 hours under the same incubation conditions after being inoculated in 5 mL of brain heart infusion (Himedia Laboratories, Mumbai, India). To achieve a concentration of 1.5 x 108 CFU/ml (a suspension comparable to a 0.5 McFarland standard), microbial cells were diluted with distilled water. One milliliter of each irrigation solution was added to one milliliter of each organism suspension. One hundred microliters of each mixture were then extracted at intervals of three, five, and ten minutes. To find the number of colony-forming units (CFU) per plate, the sample was plated on Brain Heart Infusion agar (Himedia Laboratories, Mumbai, India) following each contact period. For every contact interval, the number of CFU/mL was computed based on the average number of CFUs in the three bacterial growth zones on each plate. After being cultivated in brain heart infusion (BHI) broth at 37°C for an entire night, the culture was injected in Mueller-Hinton agar plates and adjusted to a turbidity value of 0.5 on the McFarland scale (1.5 x 108 bacteria/ml). The antibacterial inhibition zones surrounding test irrigants were identified using the agar disc diffusion method. Each medication was put to its corresponding well on BHI agar plates. For a whole day, the plates were kept in an incubator at 37°C. Using IBM USA's SPSS statistical software version 25.0, the collected data was statistically assessed using the ANOVA test P<0.05.

## Results:

No bacterial growth of *E. faecalis* or *S. Aureus* was observed in nanoparticles irrigant group and Herbal groups. While control group showed normal bacterial growth (Table 1, 2 - see PDF). The difference was statistically significant (P<0.05). Highest inhibitory zone was found with nanoparticle based irrigants followed by *triphala* and turmeric irrigants. Lowest inhibitory zone was found with control group (Table 3 - see PDF).

## Discussion:

Many bacterial species are known to play a major role in the development of pulp and periapical disorders [5]. Because it is one of the most commonly isolated species in recurrent root canal infections. *E. faecalis*, a facultative Gram positive anaerobic coccus, was selected for the current investigation. Additionally, this bacterium can withstand severe environments with limited nutrition, and it can survive longer in root canals that have been treated. It can withstand extremely high pH, acidity, and heat. Staph Aureus was chosen because it is another microbe that frequently causes primary endodontic infections and treatment failures [2]. Since mechanical techniques are insufficient to eradicate all germs, proper irrigation along with intracanal medicine is recommended. The search for alternative herbal medicines has been prompted by the ongoing rise of antibiotic- resistant strains and the negative effects of chemical irrigants. This study tested alternative herbal and silver-based nanoparticle-based irrigants. We discovered that both work well against root canal infections. The best root canal irrigants should be nontoxic, biocompatible, and have an acceptable flavor and taste [11]. Due to pharmacological failure, unpleasant reactions, pharmaceutical costs, and bacterial resistance to antimicrobials, the use of medicinal plants as medicine has grown globally [12]. The antibacterial efficacy of chlorhexidine and herbal root canal irrigants against *Enterococcus faecalis* was evaluated by Bhavani *et al.* They came to the conclusion that *Aloe vera* and *triphala* showed an inhibitory zone against *E. faecalis*. Compared to *Aloe vera*, they discovered a greater zone of inhibition with *triphala* [7]. Herbal irrigants are effective against *E. faecalis*, according to Srikumar *et al.*'s comparison of sodium hypochlorite 5%, *triphala*, and 2% chlorhexidine gluconate solutions [13]. Using sodium hypochlorite (NaOCl), Rodrigues *et al.* assessed the antibacterial properties of herbal root canal irrigants (*Morindacitrifolia*, *Azadirachta indica extract*, *Aloe vera*). They came to the conclusion that herbal remedies (*M. citrifolia*, *A. Vera* and *A. indica* extract) demonstrated an inhibitory zone against *E. faecalis* [10]. When compared to 2% CHX, Rathee *et al.* found that herbal medicines (neem, tulsi) demonstrated considerable antibacterial efficacy in primary endodontic infections [14]. Moghadas *et al.* compared the antibacterial effectiveness of an endodontic irrigation solution based on nanosilver particles to 5.25% NaOCl. They came to the conclusion that nanosilver particles work just as well as NaOCl to stop common root canal bacteria from growing [2].

The antibacterial effectiveness of several irrigants in combination with silver nanoparticles was assessed by Chhabra *et al.* In comparison to the other examined groups, they found that the combination of sodium hypochlorite and silver nanoparticles demonstrated greater antibacterial efficiency [1]. According to Bhandi *et al.*'s systematic study, the effectiveness of silver nanoparticles as endodontic irrigants depends on the size of the particles and the length of contact [5]. Compared to traditional endodontic irrigants, nanoparticle-based disinfection methods have a number of advantages, especially in terms of biofilm penetration and long-term antibacterial activity [15]. In order to eradicate *Enterococcus faecalis* (*E. faecalis*) bacteria from infected primary root canals, Shalan *et al.* compared the antibacterial efficacy of two herbal extracts with 2.5% sodium hypochlorite and saline. They came to the conclusion that all herbal irrigation solutions effectively eliminated *E. faecalis* and may be used in place of NaOCL when irrigating primary teeth's infected root canals [16]. Irrigant based on nanosilver that specifically targets certain microorganisms in the root canal space. Silver nanoparticles have intriguing properties that might make them perfect for root canal irrigation. The bactericidal action is actually mediated by the physiologically active silver ions released by silver and nanosilver in aqueous solution [17]. Smaller silver particles that are less harmful to human cells can now be produced thanks to nanotechnology. The peptidoglycan cell wall and plasma membrane, cytoplasmic DNA, and bacterial proteins are the three primary parts of the bacterial cell that interact with silver ions to create the bactericidal action [18]. Numerous researches have also demonstrated the antibacterial and anti-inflammatory properties of ginger, *Aloe vera*, turmeric, and *triphala* herbal remedies [19, 20, 21-22]. Tested root canal irrigants can be used for primary and permanent teeth to eradicate the root canal microorganisms. The study's limitations include it *in vitro* design and small sample size. The requirement for fresh processing and taste tweaking for acceptability is a significant drawback of plant extracts. 

## Advancement to knowledge:

Silver nanoparticles (AgNPs) and herbal-based nanoparticle solutions have emerged as powerful, biocompatible substitutes or supplements to conventional root canal irrigants such as sodium hypochlorite (NaOCl) and chlorhexidine (CHX). These developments provide strong antibacterial activity against resistant bacteria, especially *Enterococcus faecalis* and are frequently made utilizing "green chemistry" (plant extracts).

## Conclusion:

Root canal irrigants based on silver nanoparticles were successful in preventing *E. faecalis* from flowing through herbal irrigants. Thus, it can be used as a root canal irrigant for both primary and permanent teeth in place of sodium hypochlorite.
